# P35 A retrospective study of antimicrobial stewardship in adult patients with *Clostridioides difficile* infections 2023–24

**DOI:** 10.1093/jacamr/dlaf118.042

**Published:** 2025-07-14

**Authors:** Matthew Youngman, Shumi Matereke, Philippa King, Beverley Palmer

**Affiliations:** West Suffolk Hospital, Bury St Edmunds, UK; West Suffolk Hospital, Bury St Edmunds, UK; West Suffolk Hospital, Bury St Edmunds, UK; West Suffolk Hospital, Bury St Edmunds, UK

## Abstract

**Background:**

Increasing *Clostridioides difficile* infections (CDIs) nationally poses concern for patient safety and for limited bed capacities in acute Trusts. At West Suffolk Hospital (WSH), there was a total of 97 CDI cases in 2023/24, 48 cases over our Trust’s limit.

**Objectives:**

Whilst the increase is likely multifactorial, we sought to investigate behaviours in prescribing practice associated with antibiotics, proton pump inhibitors (PPIs) and laxatives, in primary care and secondary care that could be associated with potential risk of CDI.

**Methods:**

Patients were identified from the WSH infection prevention control teams rolling data base of Community Onset Healthcare Associated (COHA) and Healthcare Onset Healthcare Associated (HOHA) cases. All available 61 cases out of 97 for the 2023/24 financial year were analysed. This was a single-centre retrospective observational study with convenience sampling. System One and e-Care (Cerner EPMA) were utilized for data extraction.

**Results:**

55/61 (90%) were >65 years of age and 12/61 (20%) were from a care home. 56/61 patients (91.8%) received antibiotics from a GP 4 weeks prior to a CDI toxin positive result, or as an inpatient or both. 5/61 patients (8.2%) did not receive antibiotics from either primary or secondary care. 16/61 (26.2%) of patients prescribed antibiotics from their GP in primary care, 12/16 (75%) were narrow-spectrum and only 1/16 (6.25%) had course lengths >7 days. In contrast, 52/61 (85%) were prescribed antibiotics as an inpatient, 88.4% of these were on broad-spectrum antibiotics. From the group that had antibiotics prescribed as an inpatient but not by their GP 40/61 (65.5%), 15/40 (37.5%) piperacillin-tazobactam prescriptions, 6/15 (40%) had course lengths >7 days, predominantly in aspiration pneumonia (3/15, 20%). 14/40 (35%) that received multiple broad-spectrum antibiotics, 7/14 (50%) were >7 days, 4/14 (28%) were sepsis of unknown origin. 16/61 (26.2%) were prescribed laxatives in the last 4 weeks by their GP and/or as an inpatient. 8/16 (50%) of these were prescribed laxatives as an inpatient, 78% of patients refused them. Only 6/16 (37.5%) of inpatients had their laxatives stopped on identification of CDI toxin. 5/16 (31.3%) of patients who received laxatives as an inpatient had a stool sample taken after laxative administration. PPIs were prescribed by GPs in 12/31 (39%) of cases and 15/31 (49%) of patients had PPIs prescribed as an inpatient. On receipt of a CDI toxin positive result, only 34.5% had their PPI stopped, where the PPI was continued this was only appropriate in 15.7% of cases. Of the 20 repeat samples (19.4% of the 97 CDI cases), 2/20 (10%) were recurrence and 18/20 (90%) were relapse.

**Conclusions:**

Sepsis of unknown origin and aspiration pneumonia pose the largest antimicrobial burden for course lengths >7 days at WSH. Targeted AMS ward rounds and penicillin allergy de-labelling are methods to reduce broad-spectrum antibiotics. Rationalization of who can take stool samples within the EPMA system will help reduce inappropriate sampling. From the 20 repeat samples, 18/20 (90%) of those were deemed relapses of CDI, therefore WSH will implement the fidaxomicin EXTEND protocol.

